# Supplementary material for: Sirtuin 1, Visfatin and IL-27 Serum Levels of Type 1 Diabetic Females in Relation to Cardiovascular Parameters and Autoimmune Thyroid Disease
Source: Biomolecules. 2021 Jul 28;11(8):1110. doi: 10.3390/biom11081110 (PMC8391548; doi:10.3390/biom11081110)
Supplement: Supplementary file 1 [file biomolecules-11-01110-s001.zip › biomolecules-1299566-supplementary.pdf]

Supplementary Data

Sirtuin 1, Visfatin and IL-27 Serum Levels of Type 1 Diabetic Female in Relation to Cardiovascular Parameters and Autoimmune Thyroid Disease

Magdalena Łukawska-Tatarczuk[1.2], Edward Franek[1.5], Leszek Czupryniak[2], Ilona Joniec-Maciejak[3], Agnieszka Pawlak[4.5], Ewa Wojnar[3], Jakub Zieliński[6], Dagmara Mirowska-Guzel[3], Beata Mrozikiewicz-Rakowska[2*]

[1] *Clinical Department of Internal Diseases. Endocrinology and Diabetology. Central Clinical Hospital of the Ministry of Interior and Administration. Wołoska 137. 02-507 Warsaw. Poland; edward.franek@cskmswia.pl (E.D.); magdalukawska89@gmail.com (M. Ł-T.)*
[2] *Department of Diabetology and Internal Diseases. Medical University of Warsaw. Banacha 1a. 02-097 Warsaw. Poland; bigosik@poczta.onet.pl (L.C.)*
[3] *Department of Experimental and Clinical Pharmacology. Centre for Preclinical Research and Technology (CePT). Medical University of Warsaw. Banacha 1b. 02-097 Warsaw. Poland; Ilona.joniec@wum.edu.pl (I.J-M.); ewojnar@wum.edu.pl (E.W.); dagmara.mirowska-guzel@wum.edu.pl (D.M-G.)*
[4] *Department of Invasive Cardiology. Central Clinical Hospital of the Ministry of Interior and Administration. Wołoska 137. 02-507 Warsaw. Poland; a.pawlak1@wp.pl (A.P.)*
[5] *Mossakowski Medical Research Centre. Polish Academy of Sciences. Adolfa Pawińskiego 5. 02-106 Warsaw. Poland;*
[6] *Interdisciplinary Centre for Mathematical and Computational Modeling. University of Warsaw. Adolfa Pawińskiego 5A. 02-106 Warsaw. Poland; j.zielinski@icm.edu.pl (J.Z.)*
**Correspondence. rakowskab123@gmail.com (B.M-R.). tel. +48-22 599 25 83*

Table S1. Correlation between sirtuin 1 serum levels and selected cardiovascular (CV) and thyroid parameters.

	T1DM (n=50)		T1DM +HD (n=28)		T1DM -HD (n=22)		controls (n=30)	
**Classical risk factor of CV**								
	**Rho**	**p**	**Rho**	**p**	**Rho**	**p**	**Rho**	**p**
**BMI (kg/m2)**	-0.168	0.250	-0.106	0.590	-0.265	0.233	0.015	0.936
**Diabetes duration (years)**	-0.023	0.876	-0.112	0.570	-0.165	0.462	-	-
**Daily insulin dose (units)**	-0.209	0.146	-0.255	0.190	-0.094	0.698	-	-
**HbA1c %**	-0.266	0.065	-0.266	0.171	-0.341	0.121	0.317	0.089
**Total cholesterol (mg/dL)**	0.047	0.749	0.165	0.400	-0.154	0.495	0.184	0.330
**LDL-C (mg/dL)**	-0.021	0.886	0.024	0.903	-0.201	0.369	0.184	0.331
**HDL-C (mg/dL)**	0.257	0.075	0.305	0.115	0.177	0.431	0.047	0.804
**Triglyceride (mg/dL)**	-0.303	0.034	-0.187	0.340	-0.489	0.021	0.254	0.176
**Thyroid parameters**								
**TSH uIU/L**	-0.133	0.357	-0.118	0.550	-0.162	0.470	0.195	0.303
**ft4**	0.041	0.779	0.060	0.763	-0.137	0.545	0.256	0.172
**fT3**	0.092	0.526	-0.020	0.920	0.234	0.294	0.002	0.993
**aTPO IU/ml**	0.058	0.689	-0.111	0.575	0.075	0.740	0.051	0.788
**aTG IU/ml**	0.23	0.108	0.235	0.229	0.032	0.891	-0.198	0.295
**Thyroid volume (ml)**	0.285	0.045	0.511	0.005	-0.051	0.822	-0.100	0.597
**Selected CV parameters**								
**ECG-HR**	-0.076	0.599	0.199	0.311	-0.506	0.016	0.332	0.073
**ECG-Qtc**	0.059	0.685	0.334	0.082	-0.435	0.043	0.510	0.004
**cIMT (mm)**	0.071	0.623	-0.138	0.483	0.193	0.389	-0.052	0.783
**LAVI ml/m2**	-0.025	0.862	0.046	0.816	0.062	0.785	0.114	0.549
**LVMI g/m2**	-0.084	0.560	-0.164	0.403	0.168	0.455	-0.057	0.765
**PWD**	-0.287	0.044	-0.522	0.004	-0.049	0.827	-0.111	0.560
**RWT**	-0.260	0.069	-0.451	0.016	-0.032	0.886	-0.039	0.838
**E**	-0.220	0.125	-0.108	0.583	-0.440	0.040	-0.225	0.233
**E/A**	0.060	0.677	0.035	0.861	0.183	0.416	-0.231	0.220
**A'spt**	-0.185	0.197	0.029	0.885	-0.478	0.024	0.391	0.033
**E'spt**	-0.021	0.883	0.101	0.608	-0.202	0.369	-0.152	0.424
**E/E'spt**	-0.213	0.137	-0.205	0.296	-0.115	0.612	-0.214	0.256
**E'/A'spt**	0.189	0.189	0.119	0.545	0.426	0.048	-0.335	0.070
**DT (ms)**	0.020	0.893	0.255	0.191	-0.354	0.106	-0.112	0.556
**IVRT (ms)**	-0.068	0.641	-0.280	0.149	0.182	0.418	-0.122	0.521
**New biomarkers**								
**Sirtuin 1 & Visfatin**	-0.095	0.512	-0.081	0.683	0.019	0.932	0.16	0.397
**Sirtuin1 & IL-27**	0.329	0.020	0.445	0.018	0.151	0.502	0.305	0.101
**Visfatin & IL-27**	0.24	0.093	0.303	0.118	0.141	0.531	0.142	0.453

Abbreviations: BMI- body mass index; HbA1c- glycated hemoglobin*;* LDL-C - low-density lipoprotein cholesterol; HDL-C- high-density lipoprotein-cholesterol; TSH- thyroid-stimulating hormone; fT4- free thyroxine; fT3- triiodothyronine; aTPO-anti–thyroid peroxidase antibody; aTG- anti-thyroglobulin antibody*;* ECG-electrocardiogram*;* cIMT- carotid intima-media thickness; LAVI- left atrial volume index; LVMI- left ventricular mass index; PWD- posterior wall thickness dimension; RWT- relative wall thickness; DT- deceleration time; IVRT- isovolumetric relaxation time.

Table S2. Correlation between IL-27 serum levels and selected cardiovascular (CV) and thyroid parameters.

Variable	T1DM (n=50)		T1DM +HD (n=28)		T1DM -HD (n=22)		controls (n=30)	
**Classical risk factor of CV**								
	**Rho**	**p**	**Rho**	**p**	**Rho**	**p**	**Rho**	**p**
**BMI (kg/m2)**	-0.055	0.705	0.034	0.862	-0.228	0.307	-0.222	0.237
**Diabetes duration (years)**	0.088	0.541	0.051	0.798	-0.116	0.608	-	-
**Daily insulin dose (units)**	0.037	0.801	0.257	0.186	-0.205	0.360	-	-
**HbA1c %**	-0.048	0.739	0.049	0.805	-0.266	0.232	-0.217	0.250
**Total cholesterol (mg/dL)**	-0.217	0.13	-0.126	0.522	-0.334	0.129	-0.050	0.794
**LDL-C (mg/dL)**	-0.221	0.124	-0.122	0.537	-0.424	0.050	0.012	0.951
**HDL-C (mg/dL)**	0.049	0.737	-0.038	0.848	0.234	0.295	0.057	0.765
**Triglyceride (mg/dL)**	-0.202	0.160	-0.036	0.855	-0.437	0.042	-0.260	0.165
**Thyroid parameters**								
**TSH uIU/L**	0.150	0.298	0.096	0.626	0.232	0.299	-0.124	0.515
**ft4**	-0.053	0.715	-0.123	0.534	0.039	0.863	0.303	0.104
**fT3**	0.014	0.925	0.114	0.564	-0.119	0.599	0.002	0.993
**aTPO IU/ml**	0.104	0.471	0.007	0.971	-0.260	0.243	0.051	0.788
**aTG IU/ml**	0.331	0.019	0.243	0.213	0.408	0.066	-0.194	0.303
**Thyroid volume (ml)**	0.279	0.050	0.482	0.009	0.084	0.709	0.021	0.913
**Selected CV parameters**								
**ECG-HR**	0.015	0.919	0.078	0.692	0.125	0.579	0.279	0.135
**ECG-Qtc**	-0.139	0.336	0.083	0.673	-0.411	0.057	0.248	0.186
**cIMT (mm)**	-0.046	0.752	-0.138	0.483	0.193	0.389	0.089	0.641
**LAVI ml/m2**	0.072	0.620	0.206	0.294	0.114	0.612	0.096	0.614
**LVMI g/m2**	-0.031	0.832	-0.091	0.645	0.138	0.539	-0.182	0.336
**PWD**	-0.148	0.306	-0.371	0.052	0.043	0.848	-0.193	0.306
**RWT**	-0.194	0.178	-0.389	0.041	-0.148	0.510	0.054	0.779
**E/A**	0.126	0.383	0.148	0.451	0.317	0.151	0.217	0.249
**E/E'spt**	0.022	0.881	0.053	0.789	-0.036	0.875	-0.270	0.150
**E'/A'spt**	0.184	0.202	0.145	0.462	0.334	0.129	0.151	0.425
**DT (ms)**	0.140	0.333	0.350	0.068	-0.071	0.754	0.079	0.677
**IVRT (ms)**	-0.224	0.119	-0.256	0.189	-0.309	0.162	0.181	0.339

Abbreviations: BMI- body mass index; HbA1c- glycated hemoglobin*;* LDL-C - low-density lipoprotein cholesterol; HDL-C- high-density lipoprotein-cholesterol; TSH- thyroid-stimulating hormone; fT4- free thyroxine; fT3- triiodothyronine; aTPO-anti–thyroid peroxidase antibody; aTG- anti-thyroglobulin antibody*;* ECG-electrocardiogram*;* cIMT- carotid intima-media thickness; LAVI- left atrial volume index; LVMI- left ventricular mass index; PWD- posterior wall thickness dimension; RWT- relative wall thickness; DT- deceleration time; IVRT- isovolumetric relaxation time.

Table S3. Correlation between visfatin serum levels and selected cardiovascular (CV) and thyroid parameters.

Variable	T1DM (n=50)		T1DM +HD (n=28)		T1DM -HD (n=22)		controls (n=30)	
**Classical risk factor of CV**								
	**Rho**	**p**	**Rho**	**p**	**Rho**	**p**	**Rho**	**p**
**BMI (kg/m2)**	-0.071	0.625	-0.060	0.763	-0.139	0.538	0.370	0.044
**Diabetes duration (years)**	-0.007	0.961	-0.086	0.665	-0.011	0.960	-	-
**Daily insulin dose (units)**	0.001	0.996	0.068	0.732	-0.008	0.973	-	-
**HbA1c %**	0.029	0.839	-0.088	0.658	-0.143	0.524	-0.243	0.196
**Total cholesterol (mg/dL)**	-0.297	0.036	-0.375	0.049	-0.336	0.126	0.144	0.449
**LDL-C (mg/dL)**	0.171	0.234	-0.157	0.425	-0.239	0.284	0.352	0.057
**HDL-C (mg/dL)**	-0.221	0.123	-0.268	0.167	-0.205	0.360	-0.298	0.110
**Triglyceride (mg/dL)**	0.099	0.492	-0.057	0.774	0.264	0.234	0.041	0.830
**Thyroid parameters**								
**TSH uIU/L**	-0.018	0.899	-0.182	0.354	0.157	0.484	0.064	0.737
**ft4**	-0.240	0.093	-0.203	0.300	-0.591	0.004	0.238	0.205
**fT3**	-0.061	0.676	0.114	0.564	-0.187	0.405	-0.013	0.944
**aTPO IU/ml**	0.321	0.023	0.093	0.636	0.299	0.177	0.093	0.627
**aTG IU/ml**	0.137	0.344	0.119	0.546	-0.193	0.402	0.141	0.456
**Thyroid volume (ml)**	0.149	0.303	0.319	0.098	0.084	0.709	-0.019	0.919
**Selected CV parameters**								
**ECG-HR**	0.163	0.257	0.124	0.528	0.254	0.255	-0.266	0.156
**ECG-Qtc**	-0.065	0.653	0.001	0.996	-0.164	0.466	-0.076	0.689
**cIMT (mm)**	-0.068	0.639	-0.246	0.207	-0.050	0.825	0.447	0.013
**LAVI ml/m2**	0.165	0.251	0.275	0.156	0.136	0.548	-0.027	0.889
**PWD**	0.100	0.490	0.095	0.629	-0.049	0.827	0.073	0.703
**LVMI g/m2**	0.010	0.947	0.235	0.229	-0.161	0.473	-0.090	0.635
**RWT**	0.125	0.388	0.073	0.713	-0.027	0.906	0.165	0.384
**E/A**	0.013	0.931	0.020	0.919	0.057	0.802	0.144	0.448
**E/E'spt**	-0.071	0.626	-0.095	0.631	-0.141	0.531	0.127	0.505
**E'/A'spt**	0.163	0.259	0.270	0.165	0.242	0.277	0.059	0.755
**DT (ms)**	0.066	0.649	0.107	0.588	0.066	0.770	0.424	0.019
**IVRT (ms)**	0.027	0.853	-0.001	0.995	-0.029	0.898	0.340	0.066

Abbreviations: BMI- body mass index; HbA1c- glycated hemoglobin; LDL-C - low-density lipoprotein cholesterol; HDL-C- high-density lipoprotein-cholesterol; TSH- thyroid-stimulating hormone; fT4- free thyroxine; fT3- triiodothyronine; aTPO-anti–thyroid peroxidase antibody; aTG- anti-thyroglobulin antibody*; ECG*-electrocardiogram*;* cIMT- carotid intima-media thickness; LAVI- left atrial volume index; LVMI- left ventricular mass index; PWD- posterior wall thickness dimension; RWT- relative wall thickness; DT- deceleration time; IVRT- isovolumetric relaxation time.
